# THE NEEDS OF FAMILY CAREGIVERS OF PERSONS LIVING WITH DEMENTIA CARED FOR IN PRIMARY CARE PRACTICES

**DOI:** 10.1093/geroni/igad104.1768

**Published:** 2023-12-21

**Authors:** Michael Vetter

**Affiliations:** Brandeis University, Waltham, Massachusetts, United States

## Abstract

**Background:**

The proportion of persons living with dementia (PLWD) in the US is projected to increase; the majority will be cared for by family caregivers and primary care providers. Purpose: At baseline for intervention studies to support primary care practices caring for persons with dementia, we assessed caregiver self-efficacy to inform our interventions.

**Methods:**

We conducted a survey of 143 caregivers of persons living with dementia receiving care from 34 primary care practices in a Medicare ACO. The survey was conducted by postal mail and telephone in English and Spanish and used 9-item caregiver self-efficacy scale.4 , analyzing variation by caregiver relationship and location. Results A majority of 143 respondents were female (70%), White Non-Hispanic (80.4%) and lived with person with dementia (65.7%). Caregivers agreed that their doctors understand how memory or behavioral problems complicate health (85.3%) and least likely to agree that they had a health professional who helped them work through dementia care problems (39.2%). Only 45% agreed that they received advice on what problems to expect in the future related to Alzheimer’s or dementia. Caregivers who lived with the care recipient were significantly less likely than those who did not to report getting advice about common behavioral problems (43.6% vs. 68.6%, P = .005) and how to access community services (63.8% vs. 81.3%, P = .03). Discussion System innovations to support caregivers should tailor information to help caregivers of and PLWD to prepare for course of illness including treatment and management of behavioral symptoms of dementia.

